# Human BM-MSC secretome enhances human granulosa cell proliferation and steroidogenesis and restores ovarian function in primary ovarian insufficiency mouse model

**DOI:** 10.1038/s41598-021-84216-7

**Published:** 2021-02-25

**Authors:** Hang-soo Park, Rishi Man Chugh, Abdeljabar El Andaloussi, Elie Hobeika, Sahar Esfandyari, Amro Elsharoud, Mara Ulin, Natalia Garcia, Mahmood Bilal, Ayman Al-Hendy

**Affiliations:** 1 Department of Obstetrics and Gynecology, University of Chicago, 5841 S. Maryland Ave., Chicago, IL 60637 USA; 2 Department of Surgery, College of Medicine, University of Illinois at Chicago, Chicago, IL 60612 USA; 3 Department of Pathology, College of Medicine, University of Illinois at Chicago, Chicago, IL 60612 USA; 4 Fertility Centers of Illinois, Glenview, IL 60026 USA; 5 Rosalind Franklin University, North Chicago, IL 60064 USA

**Keywords:** Reproductive disorders, Mesenchymal stem cells

## Abstract

Primary ovarian insufficiency (POI) is defined as the loss of ovarian function before 40 years of age. It clinically manifests as amenorrhea, infertility, and signs of estrogen insufficiency. POI is frequently induced by chemotherapy. Gonadotoxic chemotherapy reagents damage granulosa cells, which are essential for follicular function and development. Our recently published studies demonstrated that intraovarian transplantation of human mesenchymal stem cells (hMSCs) can restore fertility in a chemotherapy-induced POI mouse model. However, the regenerative mechanism underlying the hMSC effect in POI mice is not fully understood. Here, we report that the hMSC secretome increased the proliferation of human granulosa cells (HGrC1). We showed by FACS analysis that treatment of HGrC1 cells with hMSC-conditioned media (hMSC CM) stimulates cellular proliferation. We also demonstrated that the expression of steroidogenic enzymes involved in the production of estrogen, CYP19A1 and StAR, are significantly elevated in hMSC CM-treated HGrC1 cells. Our data suggest that hMSC CM stimulates granulosa cell proliferation and function, which may explain the therapeutic effect of hMSCs in our chemotherapy-induced POI animal model. Our findings indicate that the hMSC secretome may be a novel treatment approach for restoring granulosa cell and ovarian function in patients with POI.

## Introduction

Primary ovarian insufficiency (POI), previously known as premature ovarian failure, is characterized by the loss of ovarian function before 40 years of age. Amenorrhea, infertility, and sigs of estrogen insufficiency are the main clinical manifestations reported in affected individuals. POI is diagnosed based on serum follicle-stimulating hormone (FSH) levels in the menopausal range and secondary amenorrhea. Other diagnostic features suggestive of POI are low levels of anti-Mullerian hormone (AMH) level and circulating estrogen^1–3^. Chemotherapy with gonadotoxic regimens can cause POI, and the incidence of chemotherapy-induced POI is increasing worldwide due to the increase in incidence of cancer in younger individuals. Hence, many women of reproductive or pre-reproductive age develop POI as a result of exposure to gonadotoxic cancer therapies^4–6^.

Previous studies have suggested various approaches to the treatment of POI, most recently, the use of stem cells^5,7^. Human mesenchymal stem cells (hMSCs) are the most well-known multipotent adult stem cells. They can be isolated from various mesodermal tissue types, including bone marrow, umbilical cord blood, adipose tissue, and dental pulp. Various therapeutic uses of MSC transplantation have been documented^8^. Our group reported that intraovarian injection of hMSCs can restore fertility in a chemotherapy-induced POI mouse model, leading to restoration of serum hormone levels and folliculogenesis^8,9^. The mechanisms underlying the observed therapeutic effect of MSC transplantation are not clear. A recent study revealed that small membrane-enclosed vesicles, known as exosomes, isolated from MSCs could protect granulosa cells against chemotherapy-induced damage^10^. The authors found that MSC exosomes can stimulate the recovery of ovarian granulosa cells after chemotherapy-induced stress and apoptosis in vitro. This study focused on exosome-related anti-apoptotic effects but did not examine the effect of secreted factors of MSC on another intracellular pathways such as steroidogenesis or proliferation of ovarian granulosa cells. Moreover, the anti-apoptotic effects of MSC-derived exosomes were not examined in a POI animal model.

We hypothesize that restoration of estrogen production, ovarian volume, and fertility potential in chemotherapy-induced POI mice following intraovarian injection of MSCs is due to the paracrine effects of factors produced by the MSCs. To test this hypothesis, we examined the effect of factors secreted by MSCs on the proliferation and function of a human nonluteinized granulosa cell line (HGrC1) cultured with conditioned media from MSCs (hMSC CM). We analyzed apoptosis, proliferation, and steroidogenesis in hMSC CM-treated HGrC1 to determine the mechanism underlying the effect of MSCs in chemotherapy-induced POI mice.

## Materials and methods

### MSC cell culture and CM collection

We used MSCs purchased from Lonza (cat no: PT-2501, Switzerland), which were isolated from the bone marrow of a 24-year-old healthy female donor. MSCs (4000 cells/cm^2^) were cultured in DMEM/F12 (Gibco, Waltham, MA, USA)-based MSC culture media containing 10% FBS (Omega, Tarzana, CA, USA) and 1% penicillin/streptomycin (Gibco). For collection of CM, MSCs were cultured with DMEM/F12 media (Gibco) without serum for 24 h until reaching 80–90% confluence. hMSC CM was collected and cell debris was removed by centrifugation at 500 g for 5 min at 4 °C, and the supernatant was aliquoted and stored at -80 °C for further use. hMSCs cultured in DMEM/F12 media (Gibco) without serum were used as the negative control. For the treatment of HGrC1 cells, hMSC CM was prediluted with regular DMEM/F12 media at a 1:1 ratio, and HGrC1 cells were cultured in the prediluted media for 24 h. Total protein concentration of the hMSC CM was measured by the Bradford method. To characterize hMSC-derived exosomes, we isolated exosomes from hMSC CM with ExoQuick-TC (SBI, Palo Alto, CA). The concentration of exosomal protein was determined by the Bradford method. Size and mass of hMSC-derived exosomes were analyzed using Malvern Zetasizer Nano ZSP Dynamic Light Scattering (Malvern Panalytical, Malvern, UK).

### Human granulosa cell culture

Dr. A. Iwase (Nagoya University, Japan) kindly provided the HGrC1 immortalized human granulosa cell line. HGrC1 cells were cultured in DMEM-F12 culture medium supplemented with 10% FBS at 37 °C and 5% CO_2_. DMEM-F12 culture media and FBS were purchased from Life Technologies (Thermo Fisher Scientific Inc., MA, USA). Prior to each experiment, cells were plated into 6- or 24-well plates at a concentration of 100,000 cells per milliliter (20,000 cells/cm^2^). Cell growth was monitored, and treatment was initiated when cells reached 50% confluence.

### Inducing in vitro cellular death with cyclophosphamide

To establish an in vitro POI model, HGrC1 cells were treated with 100 µg/ml cyclophosphamide for 24 h to induce apoptosis. After HGrC1 apoptosis was induced, cells were divided into two groups for further experiments. One group was treated with regular serum-free media (Group I), and the other group was treated with a 1:1 mixture of regular serum-free media and MSC CM (Group II). After 24 h of treatment, cells were collected to analyze markers of apoptosis, proliferation, and steroidogenesis.

### Cell doubling time calculation

After chemotherapy treatment, HGrC1 cells were trypsinized and then we plated same number of cells for subsequent evaluations. After media (control) or MSC CM treatment, cells were collected using trypsin. Cells from two wells combined into a single tube before centrifugation to minimize cell loss. Cells were stained with trypan blue (Sigma, St. Louis, MO) for cell counting, and the number of cells after 48 h were compared with the number of cells that were originally plated in two wells (500,000 cells). Doubling time, or the time required for one cycle of cell division, was calculated using the following formula.

$$Doubling\;time\left( h \right) = \frac{Cell\;culture\;time\left( h \right)}{{\log_{2} \left( {\frac{Collected\;cell\;number}{{Plated\;cell\;number}}} \right)}}$$

### RT-PCR

RNA isolation was performed with TRIzol Reagent (Invitrogen) according to the manufacturer’s instructions. RNA was quantified by spectrophotometry at 260 nm with the Nanodrop 2000 (Thermo Fisher Scientific). Then, 1 µg of total RNA was reverse transcribed using the RNA to cDNA EcoDry premix (Takara bio USA, CA, USA). Real-time PCR was performed using the CFX96 PCR instrument with matched primers (Table 1) and Universal SYBR Green Supermix (Bio-Rad, Hercules, CA, USA). The following PCR parameters were used: initial denaturation cycle at 95 °C for 3 min, followed by 40 amplification cycles at 95 °C for 10 s, 56 °C for 15 s, and 72 °C for 1 min. The results are presented as the fold change in relative gene expression quantified using the delta-delta CT method.

**Table 1:** Primer list for RT-PCR.

Gene	Forward primer (5′–3′)	Reverse primer (5′–3′)
Human GAPDH	TGACATCAAGAAGGTGGTGAAGC	CCCTGTTGCTGTAGCCGTATTC
Human Bax	ATGTTTTCTGACGGCAACTTC	AGTCCAATGTCCAGCCCAT
Human Casp3	TGTTTGTGTGCTTCTGAGCC	CACGCCATGTCATCATCAAC
Human AKT1	ACCTCTGAGACTGACACCATG	CACTGGCTGAGTAGGAGAAC
Human BCL2	AACGTGCCTCATGAAATAAG	TTATTGGATGTGCTTTGCATTC
Human StAR	GCTGACGTGGGCTATTACTCC	CCGAGGTGGGTATTTGGGATG
Human CYP19A1	GGTCACCACGTTTCTCTGCT	GCAAGCTCTCCTCATCAAACCA
Human FSHR	TTCAAGAACAAGGATCCATTCC	CCTGGCCCTCAGCTTCTTAA
Human FOXL2	GCTATCAGTCCCGTCGCTTC	TTAGCAAACTCCAAGGCCACA

### Immunoblot analysis

To isolate total protein from human granulosa cells, HGrC1 cells were lysed with 1 × RIPA buffer (Cell Signaling, MA, USA) containing a protease and phosphatase inhibitor cocktail (Thermo Fisher Scientific Inc.). The cell lysate was centrifuged at 12,000 rpm for 5 min, and the supernatant was collected in separate tubes. Total protein concentration of the samples were determined by the Bradford method. For immunoblot analysis, 15 µg of protein samples were incubated with 1 × gel loading buffer, separated by SDS-PAGE (4–20%, Bio-Rad) and transferred to PVDF membranes using a Trans-blot turbo system (Bio-Rad). After protein transfer, the membrane was blocked with 1% skim milk and incubated with antibodies included in the apoptosis immunoblot cocktail (ab136812, 1:500, Abcam, Cambridge, UK) or antibodies against CYP19A1 (Abcam, ab18995, 1:500) and steroidogenic acute regulatory protein (StAR; Abcam, ab58013, 1:1000). After incubation with primary and secondary antibodies, the membrane was developed with Trident Femto Western HRP substrate (GeneTex, Irvine, CA, USA) and imaged using a ChemiDoc XRS + molecular imager (Bio-Rad). The density of each protein band was quantified using ImageJ and normalized to the density of the corresponding β-actin band.

### Measurement of estradiol

To measure estradiol levels in HGrC1 CM, cells were cultured with DMEM/F12 media (Gibco) without serum for 24 h until the HGrC1 cells reached 80–90% confluence. After 24 h, HGrC1 CM was collected, and cell debris was removed by centrifugation at 500 g for 5 min at 4 °C. The HGrC1 CM was sent to Rosalind Franklin University, where estrogen levels were measured by chemiluminescence immunoassay (CLIA).

### Flow cytometry

Cells were fixed and permeabilized using BD Cytofix/Cytoperm (BD Bioscience, CA, USA) according to the manufacturer’s protocol, then stained with the appropriate antibodies against the proliferation marker Ki67 (Biolegend, 350514, CA, USA), steroidogenesis pathway marker StAR (Assaypro, 32172-05171, MO, USA), and CYP19A1 (Abcam, ab18995). A total of 1 × 10^5^ cells were incubated with the antibodies for 30 min at room temperature. Cells were washed twice with PBS/2% FBS (v/v) and resuspended in PBS. A total of 10,000 cells were analyzed on a FACScan system (Becton Dickinson) and FlowJo (BD Bioscience, CA, USA) software was used for data analysis.

### Mouse model experiment

The chemotherapy-induced POI animal model and MSC intraovarian injection protocol were established as described in our previous study^8^ and is well established in the literature^11,12^. Briefly, female C57BL6 mice (6 weeks old) were treated with busulphan (30 mg/kg) and cyclophosphamide (120 mg/kg) in a single intraperitoneal injection (n = 8). The control group (n = 4) was treated with PBS. In our published work, the ovary of POI mice exhibited a reduced number of ovarian follicles and abnormal serum hormone levels at 7 days after chemotherapy. hMSCs were transplanted by intraovarian injection 7 days after chemotherapy. Prior to surgery, mice were treated with one dose of buprenorphine (0.1 mg/kg) and then anesthetized using 1 − 4% inhalation isoflurane. A small incision was made on the skin to access the ovary via the caudal abdominal cavity. The uterine horns were traced to identify the ovary. Then, 500,000 hMSCs resuspended in 10 µl of PBS were directly injected into both ovaries (MSC group, n = 4). The ovary increased in volume immediately after injection. There was no bleeding or leakage after injection in any of the mice. For the control group (n = 4), 10 µl of PBS was injected. For molecular analysis after surgery, we randomly selected 3 mice per group avoid to using unhealthy mice. The animal experimental protocol in this study was approved by the University of Illinois at Chicago Animal Care Committee (UIC ACC) and all animal experiments were performed in accordance with the ethical policy and guidelines of University of Illinois at Chicago for laboratory animals. This study was carried out in accordance with ARRIVE guidelines.

### Histological assay

Ovarian tissue was collected 2 weeks after MSC transplantation. Ovaries were placed into cassettes and fixed immediately with 10% neutral buffered formalin (10% NBF). Fixed ovaries were processed for paraffin block embedding, sectioning, and staining for H&E and immunohistochemistry (IHC) in the UIC Research Histology and Tissue Imaging Core. Ovaries from three different animals per group were analyzed to normalize cycle related variations. Primordial, primary, secondary, and antral follicles, as defined in our previous work^8^ were observed and the number of follicles at each stage was counted in three sections per ovary at least 25 µm apart (each fifth section) and that span the entire ovary. For IHC, the Ki67 antibody (Abcam, ab16667, 1:200) was used and analyzed using the algorithm of the Aperio ImageScope (Leica, Wetzlar, Germany).

### Statistical analysis

The mRNA and protein levels of the examined markers were continuous variables and expressed as means ± SDs. ANOVA and Bonferroni’s multiple comparison post hoc tests were used to compare the groups. The significance level was set at 5% (*P* < 0.05). The SPSS statistical program (version 22) was used to analyze the data.

## Results

### hMSC CM decreases cyclophosphamide-induced apoptosis of human granulosa cells

**Figure 1:**
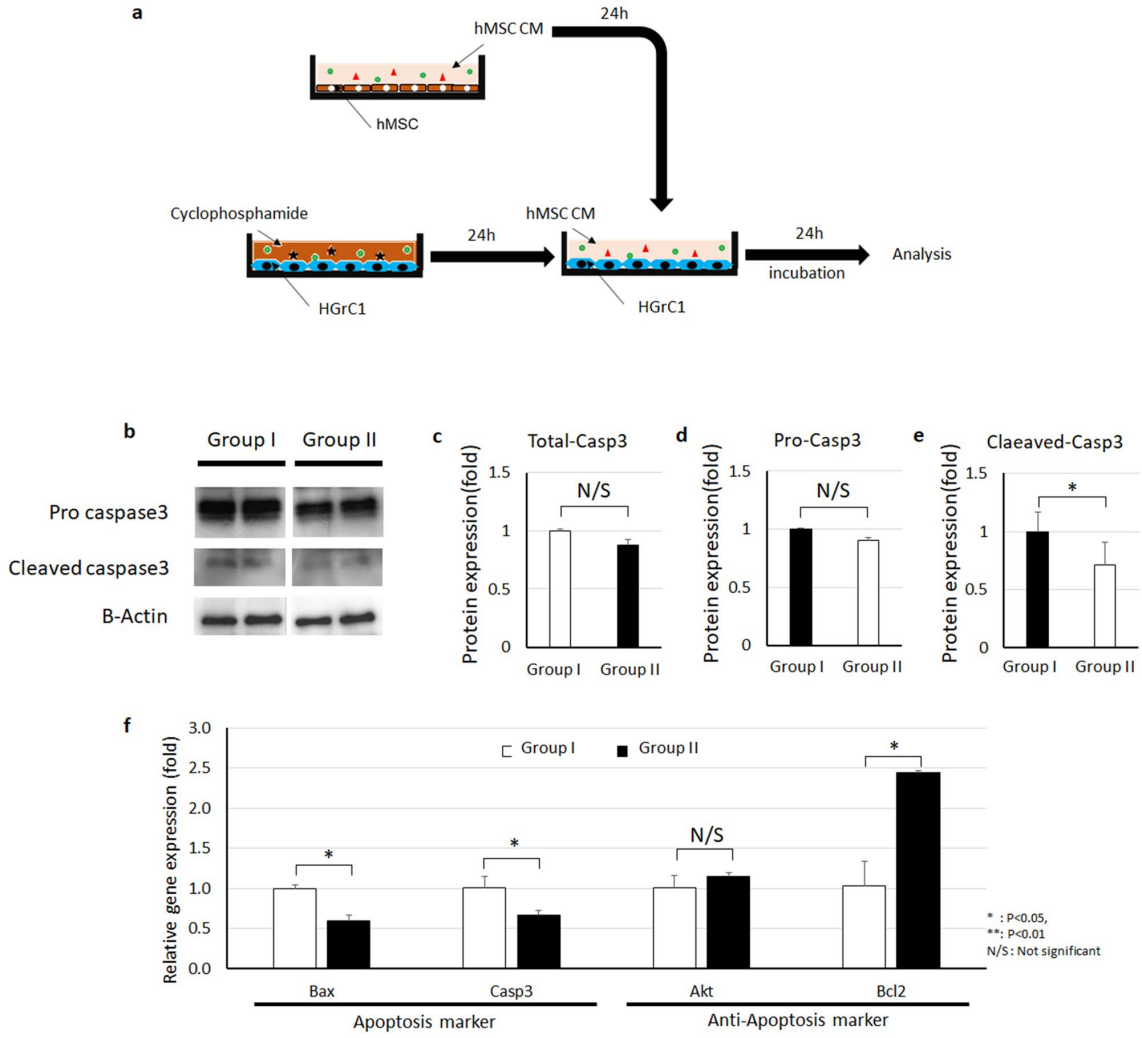
Human mesenchymal stem cell conditioned media (hMSC CM) decreases cyclophosphamide-induced apoptosis of human granulosa cells (HGrC1). (**a**) Experimental design of the in vitro study. (**b**–**e**) Expression of apoptosis marker caspase 3 by immunoblot in cyclophosphamide-treated HGrC1 that were untreated (control, Group I) or treated with hMSC CM (Group II). Quantification of total caspase 3 (**c**), pro-caspase 3 (**d**), and cleaved caspase 3 (**e**) based on immunoblot protein band density. (**f**) Apoptosis marker gene expression in cyclophosphamide-treated control HGrC1 (Group I) and hMSC CM-treated HGrC1 (Group II). Two biological replicates per treatment group were analyzed in duplicate (*P < 0.05, **P < 0.01, *N/S* not significant).

We compared apoptosis marker expression between untreated and MSC CM-treated HGrC1 cells that had been exposed to cyclophosphamide (Fig. 1a). Immunoblot analysis showed that 100 µg/ml cyclophosphamide increased cleaved caspase 3 levels in HGrC1 cells (damaged) compared to control cells (undamaged; Fig. 1b, Supplementary Figure 1). Total caspase 3 was not significantly decreased in damaged cells treated with control media (Group I) or hMSC CM (Group II; Fig. 1b–e); however, cleaved caspase 3, which was increased by cyclophosphamide (1.00 ± 0.17 fold), was significantly decreased by hMSC CM treatment (0.71 ± 0.19 fold). Our results suggest that hMSC CM can reverse markers of chemotherapy-induced apoptosis in granulosa cells. Next, we analyzed the gene expression levels of apoptosis markers by RT-PCR (Fig. 1f). Our PCR results showed that the gene expression levels of these markers, including Bax (0.59 ± 0.07-fold) and caspase 3 (0.66 ± 0.06-fold), were significantly decreased in damaged HGrC1 cells after hMSC CM treatment (Group II). Conversely, anti-apoptosis markers including Akt (1.15 ± 0.04-fold) and Bcl2 (2.44 ± 0.02-fold) increased after hMSC CM treatment (Group II). Our results demonstrate that hMSC CM can mitigate cellular damage induced by cyclophosphamide in human granulosa cells.

### hMSC CM enhances human granulosa cell proliferation

**Figure 2:**
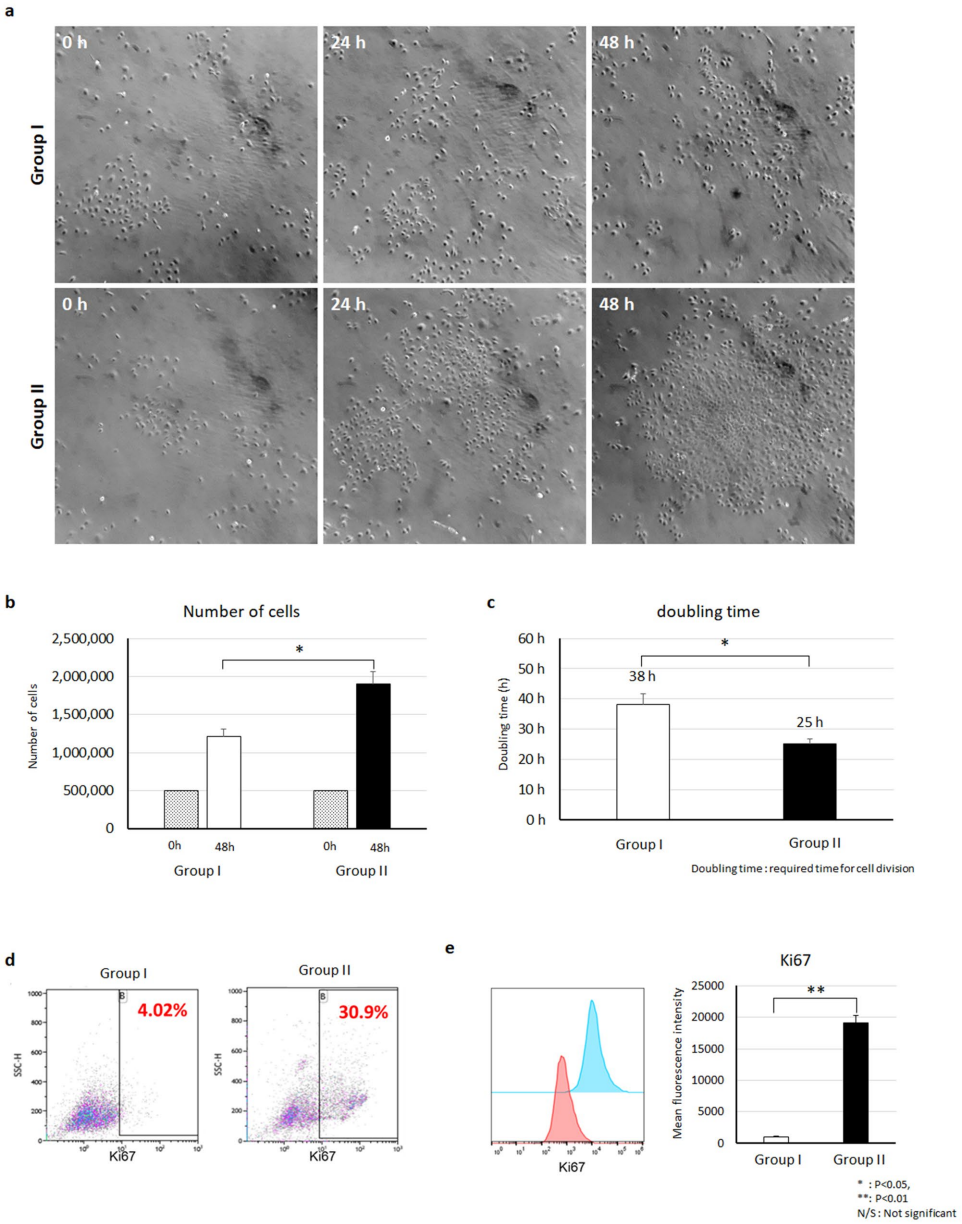
hMSC CM stimulates the proliferation of human granuloma cells. (**a**) Morphology and population changes of HGrC1 within 48 h. (**b**) Cell numbers of control HGrC1 (Group I) and MSC CM-treated HGrC1 (Group II). (**c**) Doubling times of control HGrC1 (Group I) and MSC CM-treated HGrC1 (Group II). (**d**–**e**) Flow cytometry analysis of the proliferation marker Ki67 in control HGrC1 (Group I) and MSC CM-treated HGrC1 (Group II). Two biological replicates per group were analyzed in duplicate (*P < 0.05, **P < 0.01, *N/S* not significant).

We collected HGrC1 cells after hMSC CM treatment and performed a cell count. Interestingly, we found that there were more cells in the hMSC CM-treated group than in the untreated group. We hypothesized that hMSC CM not only inhibits apoptosis but also increases the proliferation of HGrC1 cells. To measure cell proliferation, we plated the same number of cells and divided them into an untreated control group (Group I) and an hMSC CM-treated group (Group II). Cells in Group I were cultured with regular media without serum, and cells in Group II were cultured with a 1:1 mixture of regular media and hMSC CM. Analysis of changes in cell confluency showed that cells in Group II proliferated much faster than those in Group I (Fig. 2a). The number of cells in Group I after 48 h of culture was approximately 1.21 ± 0.10 × 10^6^ cells, compared to approximately 1.90 ± 0.16 × 10^6^ cells in Group II at the same point (1.57-fold higher). The average doubling time of the cells in Group I was 38 ± 4 h and in Group II was 25 ± 2 h, indicating faster cell division (Fig. 2b, c). We analyzed the proliferation marker protein Ki67 by flow cytometry (Fig. 2d, e). Approximately 31.9% of hMSC CM-treated HGrC1 cells (Group II) were positive for Ki67 compared to only 4.02% of cells in the control group (Group I). These results suggest that hMSC CM treatment increases the proliferation of human granulosa cells in vitro.

### hMSC CM stimulates steroidogenesis in human granulosa cells

**Figure 3:**
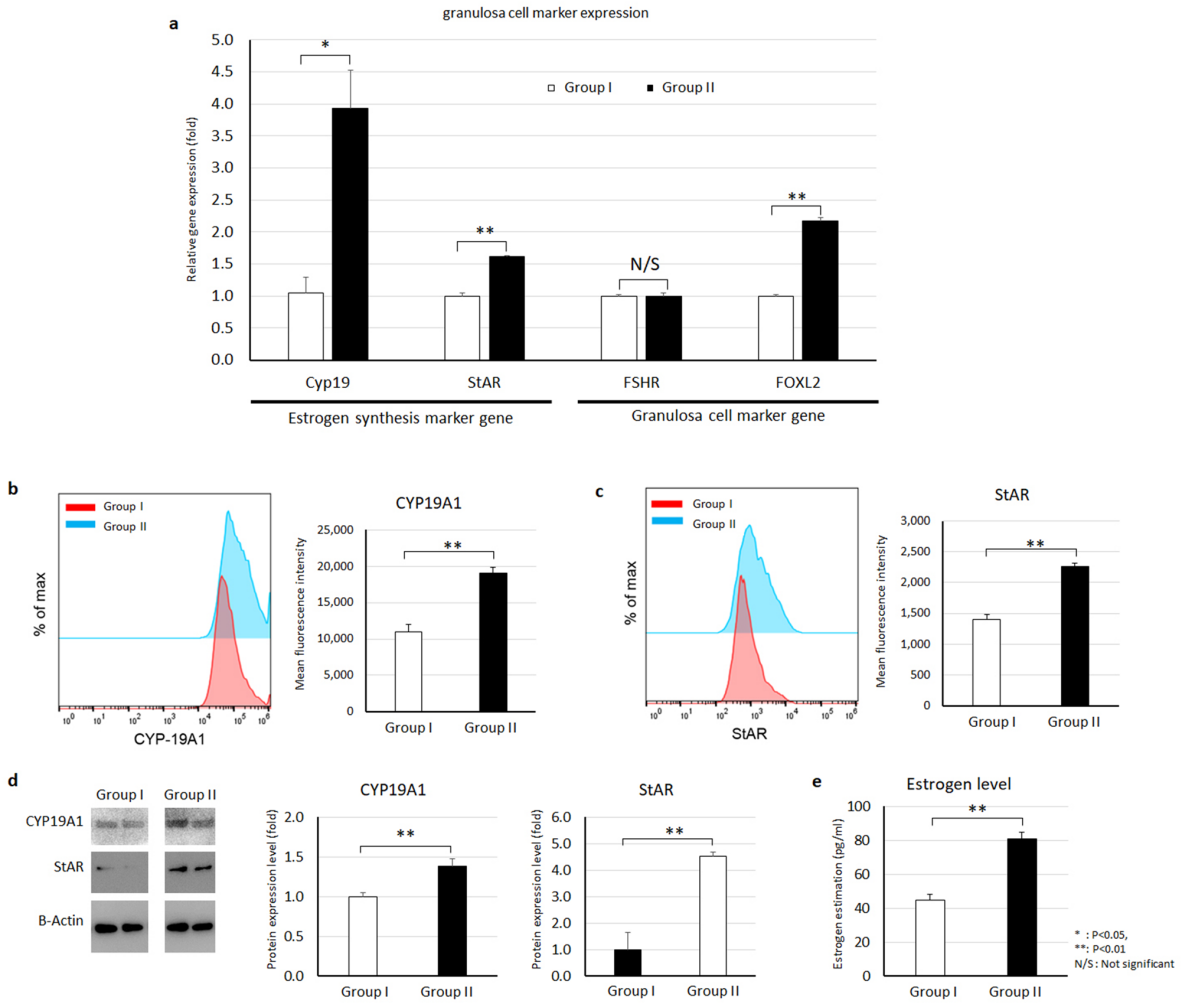
hMSC CM stimulates steroidogenesis by human granuloma cells. (**a**) RT-PCR analysis of granulosa cell marker gene expression in control HGrC1 (Group I) and hMSC CM-treated HGrC1 (Group II). (**b**–**d**) Analysis of estrogen synthesis pathway proteins CYP19A1 (aromatase) and StAR. CYP19A1 protein expression (**b**) and StAR protein expression (**c**) by flow cytometry. (**d**) CYP19A1 and StAR protein expression by immunoblot. (**e**) Analysis of estrogen levels in HGrC1 cell-cultured conditioned media. Two biological replicates per group were analyzed in duplicate (*P < 0.05, **P < 0.01, *N/S* not significant).

Next, we sought to determine the effect of hMSC CM on granulosa cell function, specifically, estrogen synthesis. We analyzed aromatase (CYP19A1) and StAR gene expression in untreated control and hMSC CM-treated HGrC1 cells. Our PCR results showed that both aromatase and StAR gene expression levels were significantly increased by hMSC CM treatment (Fig. 3a). We also measured other granulosa cell marker genes, namely, FOXL2 and FSHR. We found that the gene expression levels of FOXL2 were increased by hMSC CM; however, FSHR gene expression was not changed by hMSC CM treatment. To confirm our findings of steroidogenesis marker gene expression at the protein level, we assessed aromatase and StAR expression in cells by flow cytometry and found that the mean fluorescence intensities of aromatase and StAR were increased more than 1.5-fold after hMSC CM treatment (Fig. 3b–c). To confirm the upregulation of these proteins in hMSC CM-treated HGrC1 cells, we evaluated the same markers by immunoblot (Fig. 3d), and the results showed significantly increased expression of aromatase and StAR protein in the hMSC CM-treated group (Group II). We also measured estrogen levels in HGrC1 cell culture media (Fig. 3e). Our results showed that control HGrC1 cells (Group I) produced approximately 44.5 ± 3.5 pg/ml estrogen and hMSC CM-treated cells (Group II) produced 81.0 ± 3.6 pg/ml estrogen, a 1.8-fold increase compared to control cells. Our analysis revealed that hMSC CM can restore granulosa cell function by stimulating steroidogenesis.

### hMSCs restore cellular proliferation in the ovarian tissues of chemotherapy-induced POI mice

**Figure 4:**
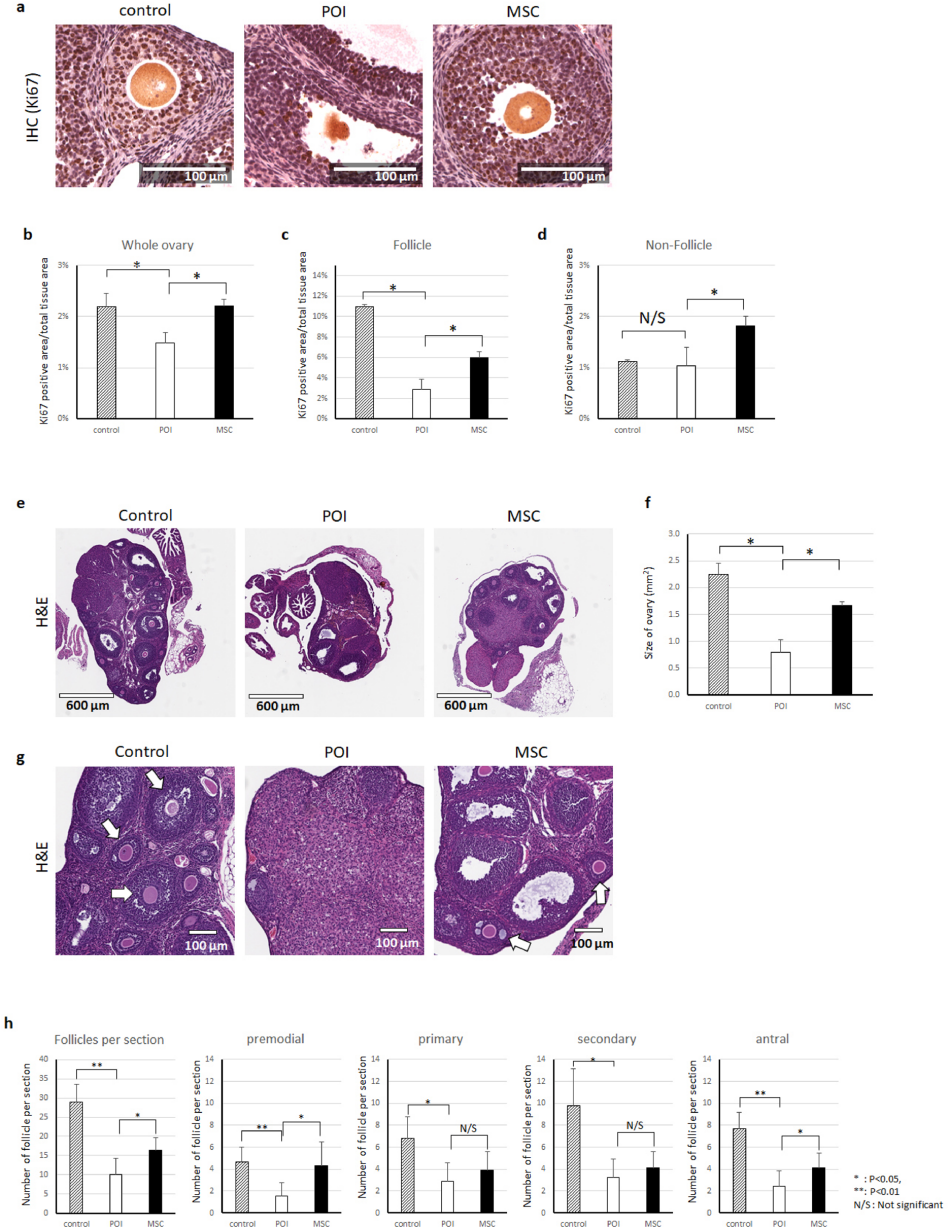
hMSCs restore proliferation in chemotherapy-induced POI ovaries. (**a**) Immunohistochemistry analysis for Ki67 in the ovary from a normal mouse (control), POI mouse (POI), and hMSC-injected POI mouse (MSC). (**b**–**d**) Comparison of Ki67-positive areas measured by immunohistochemistry. (**e**) Representative image of the whole ovary from a normal mouse (control), POI mouse (POI), and hMSC-injected POI mouse (MSC). (**f**) Comparison of the average size of the ovary in each group. (**g**) Representative images of ovarian follicles in each group. (**h**) Average number of follicles in each ovary slide between groups. Three ovaries from different animals were analyzed per group (*P < 0.05, **P < 0.01, *N/S* not significant). Images taken and analyzed using Leica Aperio ImageScope version 12.4.0.5043 (https://www.leicabiosystems.com/digital-pathology/manage/aperio-imagescope/).

Next, we sought to confirm if the observed effects of hMSC CM treatment on granulosa cells in vitro could be reproduced by hMSC injection in our animal model of POI. To this end, we established chemotherapy-induced POI in mice and transplanted hMSCs by intraovarian injection. For the control group, PBS without any cells was injected. Two weeks after hMSC transplantation, we collected and fixed the ovaries for histological analysis. We stained ovarian sections to quantify the Ki67-positive area in whole ovary sections, representing the population of proliferating cells in the ovary, using Image scopeX64 software (Fig. 4a–h). Our results showed that the Ki67-positive population in the POI mouse ovaries was significantly decreased (1.49 ± 0.20%) compared to that in the normal mouse ovaries (2.20 ± 0.25%) and that Ki67 positivity was restored in the hMSC treated group (2.20 ± 0.14%; Fig. 4b). In addition, we compared the population of Ki67-positive proliferating cells in follicular and non-follicular areas of the ovary (Fig. 4c–d). The average Ki67-positive population in follicles was approximately 10.97 ± 0.003% in the normal ovary, significantly lower in the POI ovary (2.90 ± 0.01%), and restored in the hMSC-treated ovary (5.95 ± 0.01%). The size of the POI mouse ovary was also decreased (0.79 ± 0.24mm^2^) compared to normal ovary (2.24 ± 0.22mm^2^); ovary size returned to normal levels (1.67 ± 0.07mm^2^) after hMSC treatment (Fig. 4e–f). In addition, when we counted the number of follicles, we found that the average number of follicles per ovary section was significantly higher in the hMSC-treated group (16.44 ± 3.24) compared to that in the POI group (10.11 ± 4.23) (Fig. 4g, h). This animal experiment result suggests that hMSCs restore folliculogenesis in POI mouse ovaries by stimulating ovarian cell proliferation and restoring the size of the ovary. Taken together, these data suggest that MSCs can restore the POI ovary by promoting granulosa cell viability and stimulating proliferation and steroidogenesis by secreting therapeutic factors.

## Discussion

In this study, we report that hMSC CM restores the granulosa cell population by decreasing chemotherapy-induced apoptosis and stimulating granulosa cell proliferation. In addition, we demonstrated that hMSC CM stimulates estrogen production by upregulating CYP19A1 and StAR gene expression. We previously reported that intraovarian injection of hMSC can restore fertility in a POI mouse model and engrafted hMSCs are integrated into follicle structure^8^. Our results in this study suggest that engrafted hMSCs in chemotherapy-induced POI mouse ovaries stimulate granulosa cell proliferation, which likely restores fertility as reported in our previous study^8^.

In women, chemotherapy for cancer can induce POI, primarily by cytotoxicity to ovarian granulosa cells, which induces follicular death and leads to infertility^4,6^. Many recent studies and our previous report on the therapeutic effect of hMSCs indicate that these cells may be a promising new treatment approach for patients with infertility due to chemotherapy-induced POI^5,7,8^. Granulosa cells play an important role in estrogen production and ovarian foliculogenesis^13–15^. We sought to determine whether hMSC affect granulosa cell proliferation and function through secretion of paracrine acting factors.

In many previous studies, it has been reported that chemotherapy reagent can increase apoptosis in granulosa cells^10,16,17^. We also found similar result by analyze caspase3 protein (Supplementary Figure 1a) after cyclophosphamide treatment. However, morphologically, there was no significant visible difference related with apoptosis between control group and MSC CM treated group in cell culture. Instead, we further analyzed another effect of MSC CM during cell culture such as HGrC1 cell proliferation and steroidogenic gene expression. We observed significant increase in the number of cells after 48 h of MSC CM treatment and enhanced steroidogenesis gene expression.

To analyze cell proliferation after MSC CM treatment, we compared cell number and Ki67 positive population, which represent proliferating cells^18,19^. In our result, we found that both number of cells and percentage of Ki67 expressing cells were increased after MSC CM treatment. Although we found enhanced cell proliferation through Ki67 positive population, it is still not clear which cell cycle stage is regulated by MSC CM. Analyze each cell cycle in HGrC1 cells could be an interesting topic for a further study.

In this study, we used human non luteinized granulosa cells (HGrC1) isolated from a 35-year-old female donor^20^. It is reported that HGrC1 is expressing granulosa cell marker such as FSHR and CYP19A1 while producing estrogen^20,21^. Moreover, in several published paper, HGrC1 was used as a representative in vitro model of human ovarian granulosa cell^22,23^. We cultured HGrC1 in 10% FBS until 80% of confluence in normal FBS without charcoal stripping to provide cells with abundant substrate (e.g., cholesterol) for sex steroid hormone production. Serum was removed for only 24 h during hMSC CM treatment. After 24 h of serum free condition, HGrC1 cells still show more than 90% of viability (data not shown). During that time, we expected that the steroidogenesis pathway would continue to be active until all substrate had been depleted.

The estrogen production pathway is regulated by several genes, including CYP19A1 (aromatase) and StAR^24–26^. During estrogen synthesis, StAR converts cholesterol to pregnenolone, and downstream products of pregnenolone are converted to testosterone. Eventually, testosterone is converted to estradiol by aromatase. Our study shows that hMSC CM-treated granulosa cells have increased CYP19A1 and StAR gene expression compared to untreated cells. Moreover, the estrogen level in granulosa cell conditioned media increased after treatment with hMSC CM. Thus, we demonstrated that hMSC CM can stimulate estrogen production through upregulation of CYP19A1 and StAR gene expression.

In our animal experiment, we used three groups of mice (control, POI, and POI treated with hMSC). As the ultimate goal of our work is to determine the utility of hMSCs as a treatment for POI, we treated only the POI mouse model with hMSC to compare the effect against untreated POI mice as a control. Healthy untreated mice were included as a positive environmental control to confirm the reproductive robustness of this model in our local environment. We did not treat healthy mice with hMSC, as no informative data on the therapeutic potential of hMSC would be gleaned from such an experiment, and such a treatment group had not been included in related prior work published by our group and others^8,27^.

In our POI model, we inject 10 µl of hMSC in solution directly into the ovary. The mouse ovary is extremely small, so it is technically difficult to target the bursa or any specific part of ovary, and the injected ovary appears swollen immediately after injection. There was no bleeding or leakage after injection in any of the mice. Similar to our previous study, hMSC injection did increase ovarian weight compared to non-injected animals^8^.

In our follicle counting data, we found a significant decrease in the number of follicles in POI mouse model and that the follicle number was restored after hMSC injection. To minimize the chance of counting the same follicle again, we only counted follicle with oocyte in each section. Among the types of follicles, a fixed number of primordial follicles are formed during fetal development; this pool of follicles could be expected to remain unaffected by either chemotherapy or hMSC injection in post-pubertal ovaries. Nevertheless, several previous studies showed a significant decrease in primordial follicles in chemotherapy-induced POI model animals, and restoration of this population by stem cell based therapy^27–30^. Our data also shows a significantly decreased number of primordial follicles in POI mice, and significant restoration in hMSC-treated mice. It is unclear how hMSC resume primordial follicle formation in post-pubertal ovaries. Interestingly, a recent study suggested primordial follicle activation occurs through PTEN regulation^31^, and another study revealed that a therapeutic effect of hMSC in POI involves targeting the PTEN pathway^32^. Discovering the specific signaling pathways by which transplanted hMSC stimulate the resumption of primordial follicle formation is an interesting topic for future study.

When we analyzed the number of growing follicles including primary and secondary follicles, the difference between the untreated POI group and the hMSC-treated POI group was not significant. However the proliferating cell population in growing follicles (secondary follicles) were significantly higher in the hMSC group (Supplementary Figure 3). This effect on secondary follicles was seen despite hMSC being mostly localized in smaller primary follicles. Our data suggest that hMSC treatment stimulates proliferation of granulosa cells in secondary follicles likely via a paracrine effect of growth factors secreted from injected hMSC that diffuse and reach surrounding structures within the ovary.

Other published papers have reported that MSC can restore damaged tissue by secreting therapeutic factors such as cytokines and growth factors, which as paracrine signaling factors^33,34^. Based on our in vitro experiments using cell-free hMSC CM, we conclude that the restorative effects of hMSC on the POI ovary occurs via paracrine acting factors and not via differentiation of the hMSC into ovarian cells. Recent studies have reported that exosomes may also be involved in cell-to-cell communication^35–38^. Exosomes, a specific group of extracellular vesicles (EVs), are membrane-enclosed vesicles that are secreted by cells^39–41^. Multiple recent studies have suggested that miRNAs in exosomes play important roles in ovarian granulosa cell viability^10,32,42,43^, and some studies have tried to treat POI by intraovarian injection of MSC exosomes^28,43,44^. However, the use of pure exosomes in clinical trials has several limitations. First, exosomes are rapidly degraded in vivo^45–47^, with many studies in animal models demonstrating an in vivo half-life of injected exosomes of several minutes after which they begin to degrade quickly. Another limitation of pure exosome treatment is the high exosome doses used. Many published studies have used more than 100 µg of exosomal protein in in vitro and in vivo models of POI^10,32,42,43^. Based on our experience, 100 µg of MSC-derived exosomes requires 500 ml of conditioned media using a commercially available isolation kit. To isolate pure exosomes without lipoprotein or other particles, approximately 1000 ml of conditioned media is required to collect 100 µg of exosomes^48^. This high amount of conditioned media required would increase the cost of treatment. In our study, we used whole conditioned media from cultured hMSCs and observed the same effects on reducing apoptosis and stimulating estrogen production by granulosa cells.

In our in vitro analysis, we found that 5 ml of hMSC CM contained only 0.8 µg of exosomal protein, which means we used 100-fold less than levels used in previously published papers (Supplementary Table 1). Because our hMSC CM contained other proteins, such as cytokines, it is not clear whether only 0.8 µg of pure exosomes is sufficient to enhance ovarian cell viability and estrogen production. Determination of the therapeutic dose of hMSC CM exosomes needs further research.

In this study, we demonstrate that hMSC CM treatment can enhance the viability of ovarian granulosa cells, restore ovarian structure, and stimulate estrogen production. These findings could explain, at least partially, the mechanism underlying fertility restoration in hMSC-treated POI mice reported in our previous study^8^. Based on these findings, we suggest that the paracrine acting factors secreted by hMSCs may help minimize or reverse the effects of cytotoxic chemotherapy on the ovary, hence representing a promising treatment for women with chemotherapy-induced POI.

## Supplementary Information

Supplementary Information.
