# Supplementary material for: Human BM-MSC secretome enhances human granulosa cell proliferation and steroidogenesis and restores ovarian function in primary ovarian insufficiency mouse model
Source: Sci Rep. 2021 Feb 25;11:4525. doi: 10.1038/s41598-021-84216-7 (PMC7907146; doi:10.1038/s41598-021-84216-7)
Supplement: Supplementary file 1 — Supplementary Information. [file 41598_2021_84216_MOESM1_ESM.docx]

**Human BM-MSC secretome enhances human granulosa cell proliferation and steroidogenesis and restores ovarian function in primary ovarian insufficiency mouse model**

Hang-soo Park^1ɸ^, Rishi Man Chugh^2ɸ^, Abdeljabar El Andaloussi^3ɸ^, Elie Hobeika^4^, Sahar Esfandyari^2^, Amro Elsharoud^2^, Mara Ulin^2^, Natalia Garcia^2^, Mahmood Bilal^5^, Ayman Al-Hendy^1^*

^1^Department of Obstetrics and Gynecology, University of Chicago, 5841 S. Maryland Ave. Chicago, IL 60637, USA

^2^ Department of Surgery, University of Illinois at Chicago, Medical College, Chicago, Illinois 60612, USA

^3^ Department of Pathology, University of Illinois at Chicago, Medical College, Chicago, Illinois 60612, USA

^4^ Fertility Centers of Illinois, Glenview, Illinois 60026, USA

^5^ Rosalind Franklin University, North Chicago, Illinois 60064, USA

^ɸ^ These authors equally contributed to this study

* Corresponding author:

Ayman Al-Hendy, MD, PhD, FRCSC, FACOG, CCRP

Professor

Department of Obstetrics and Gynecology

University of Chicago

5841 S. Maryland Ave.

Chicago, IL 60637

E. mail: aalhendy@BSD.Uchicago.edu

**Supplementary Figure 1**

*
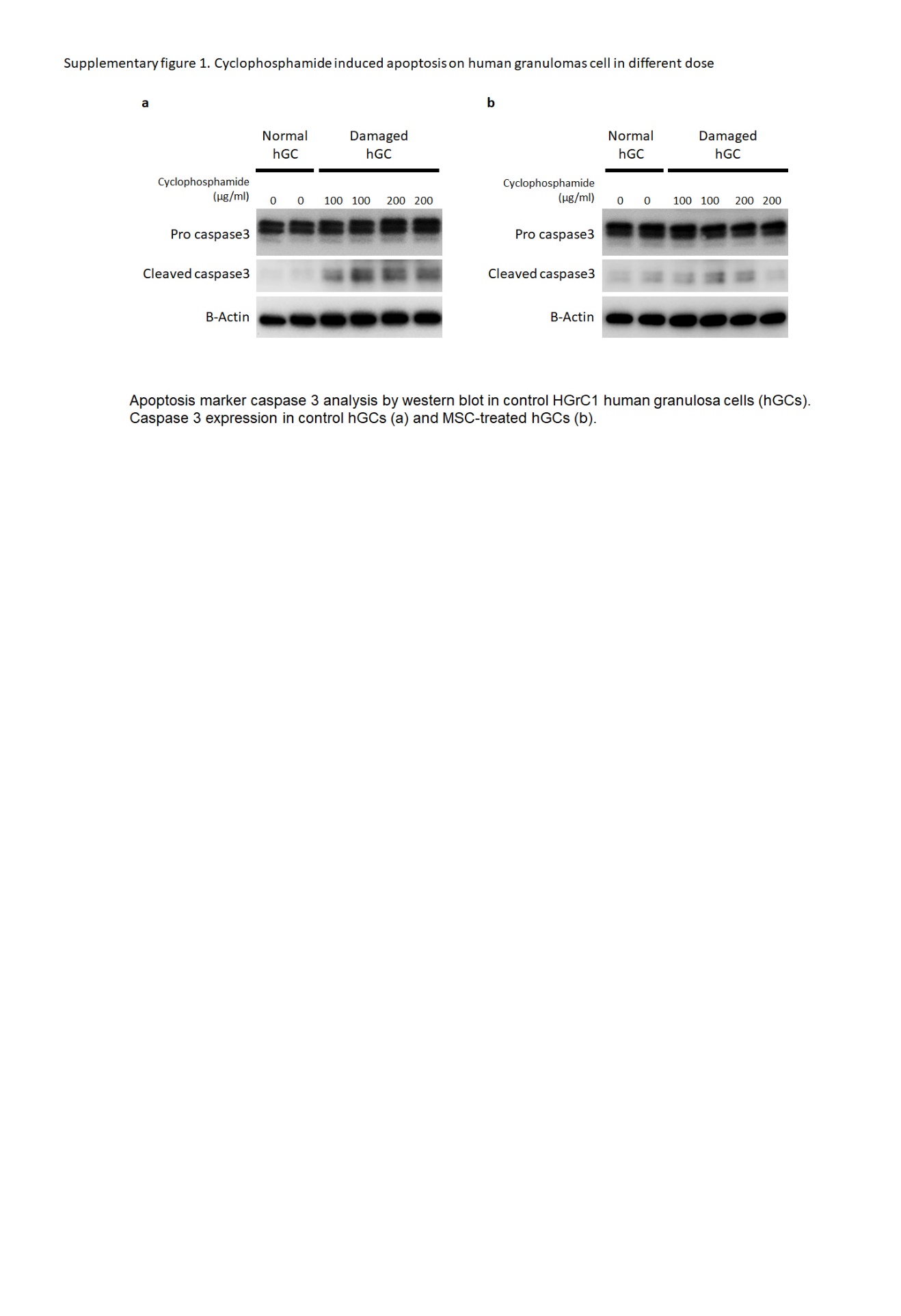
*

**Supplementary Figure 2**

*
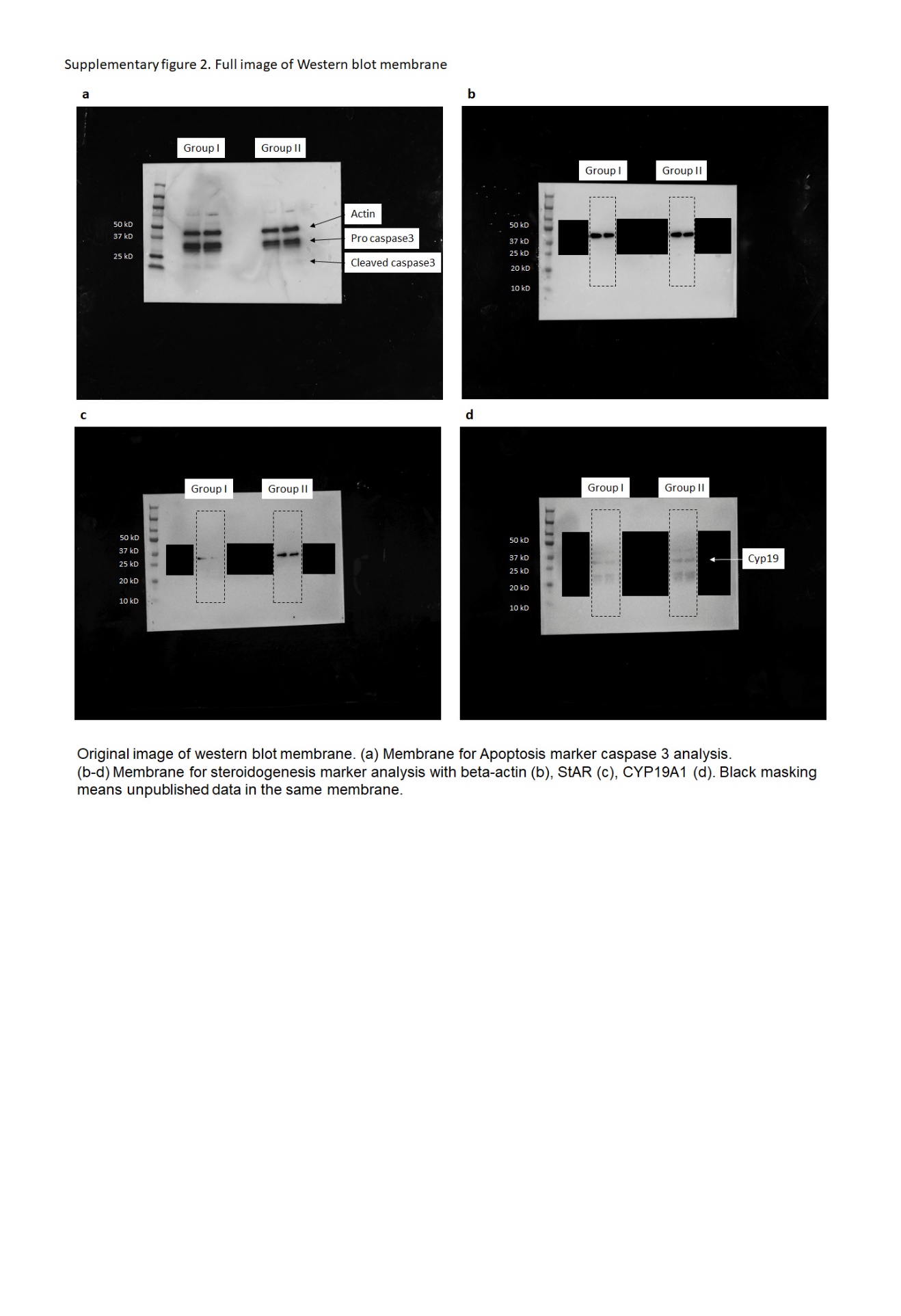
*

**Supplementary Figure 3**

*
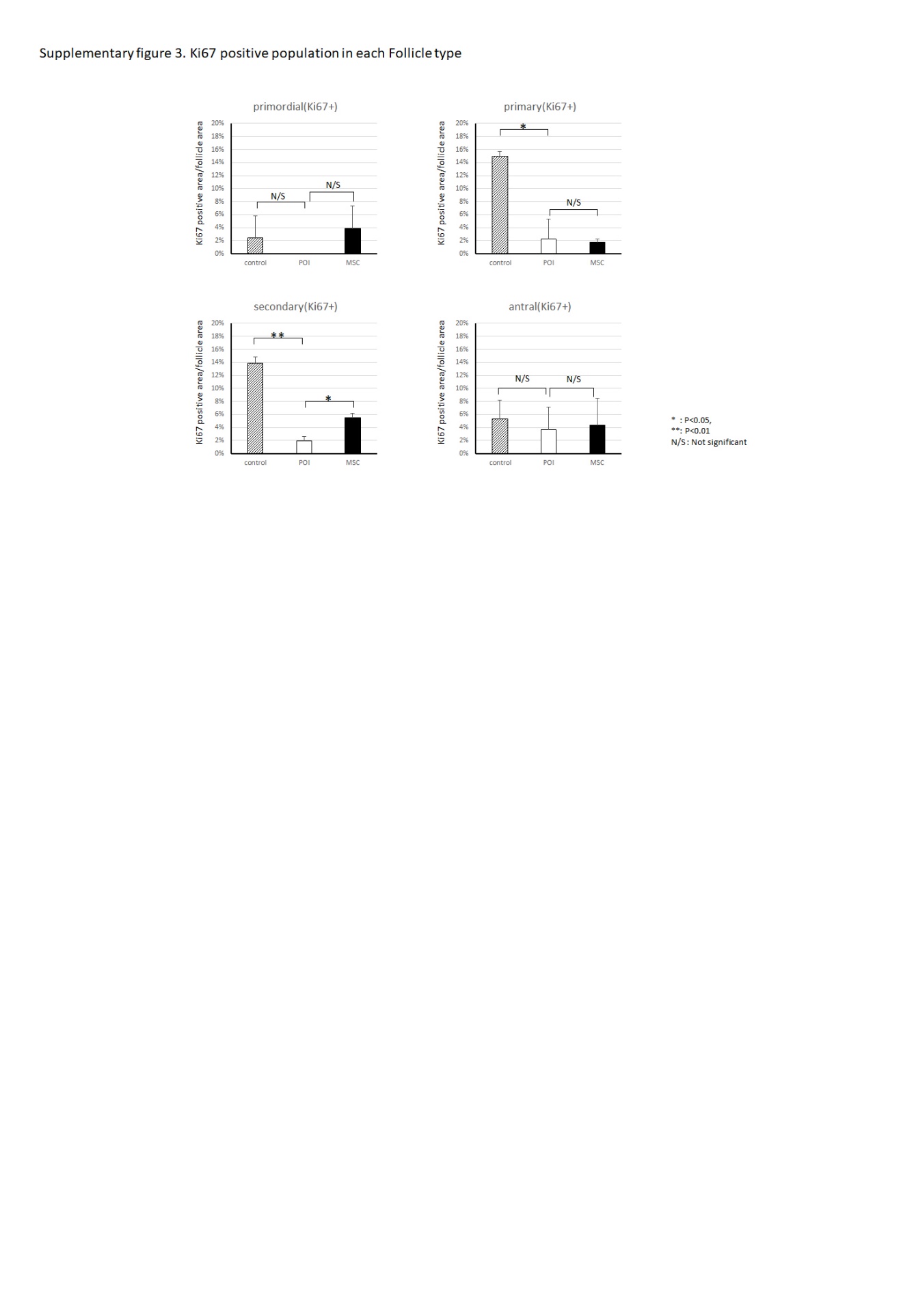
*

**Supplementary Table 1.** Characterization of the hMSC CM

|  | **Average** | **SD** |
| --- | --- | --- |
| Volume of hMSC CM (ml) | 5 ml |  |
| Number of MSCs for producing CM | 500,000 cells |  |
| Time for hMSC CM production | 24 hours |  |
| Total protein in 5 ml of hMSC CM (including other protein or cytokines) | 20.0 μg | 5.52 μg |
| Exosomes in 5 ml of hMSC CM |  |  |
| Exosome diameter | 255 nm | 21.6 nm |
| Exosome mass (kDa) | 289,000 kDa | 24,200 kDa |
| Exosomal protein | 0.8 μg | 0.17 μg |
| Number of exosome particles | 1.67 × 10^12^ | 3.54 × 10^11^ |
